# Antimicrobial resistance, characterization, and knowledge practices of *Salmonella* spp. infection in under-five children with acute gastroenteritis at Levy Mwanawasa University Teaching Hospital, Lusaka Zambia

**DOI:** 10.1093/jacamr/dlag031

**Published:** 2026-03-19

**Authors:** Namwezi Namuchimba Kapembwa, Flavien Nsoni Bumbangi, Chisanga Chipanta, Kaunda Yamba, Mike Nundwe, Misheck Shawa, John Bwalya Muma

**Affiliations:** Department of Disease Control, School of Veterinary Medicine, University of Zambia, Lusaka, Zambia; Department of Medicine and Clinical Sciences, School of Medicine, Eden University, Lusaka, Zambia; Department of Nutrition, Kinshasa School of Public Health, Faculty of Medicine, University of Kinshasa, Kinshasa, Democratic Republic of Congo; Department of Biomedical Sciences, School of Medicine, University of Zambia, Lusaka, Zambia; Department of Pathology and Microbiology, University Teaching Hospitals, Lusaka, Zambia; Department of Disease Control, School of Veterinary Medicine, University of Zambia, Lusaka, Zambia; Department of Disease Control, School of Veterinary Medicine, University of Zambia, Lusaka, Zambia; Department of Disease Control, School of Veterinary Medicine, University of Zambia, Lusaka, Zambia

## Abstract

**Bakground:**

*Salmonella* spp. is a major cause of bacterial gastroenteritis worldwide borne from consuming contaminated food or water. The growing incidence of difficult-to-treat *Salmonella* infections has been heightened by increased AMR due to increased use of antibiotics posing acritical public health challenge.

**Methods:**

This was a cross-sectional study involving 205 children with AGE at Levy Mwanawasa University Teaching Hospital in Lusaka Zambia, between September 2020 and February 2021. Stool samples were collected and subjected to standard microbiological testing, serotyping, antimicrobial susceptibility testing and molecular confirmation for *Salmonella* spp. In addition, a questionnaire was administered to participants’ guardians to determine the level of knowledge and practices towards *Salmonella* infections. Data analysis was performed using Microsoft Excel, GraphPad Prism and WHONET.

**Results:**

Twenty *Salmonella* isolates were recovered from the processed stool samples (*n* = 205), giving a 9.76% prevalence. Out of 20 *Salmonella* isolates identified, only four were susceptible to all tested antibiotics. Seven isolates (35%) were classified as multidrug resistant. The highest resistance was observed to trimethoprim-sulfamethoxazole (42.9%). Identified risk factors included use of untreated drinking water and suboptimal feeding practices.

**Conclusions:**

The presence of multidrug-resistant *Salmonella* among paediatric patients highlights the need for strengthened AMR surveillance, antimicrobial stewardship and targeted public health interventions.

## Introduction

Acute gastroenteritis (AGE) is an inflammation of the intestines causing frequently occurring diarrheal disease with symptoms such as nausea, vomiting and abdominal pain.^1^  *Salmonella* spp. commonly causes gastrointestinal infection, accounting for 93.8 million foodborne illnesses and 155 000 deaths per year.^2^ Enteric salmonellosis develops after ingestion of contaminated food or water and these infections remain restricted to the gastrointestinal tract.^3^ The most common serovar causing human illness are *Salmonella enterica* serovar Typhimurium and *Salmonella enterica* serovar Enteritidis.^4^ Identification of the pathogens responsible for infectious diarrheal diseases is crucial in surveillance, prevention and control.

Diarrhoea is the second largest cause of death in children under 5 years of age globally,^5,6^ and is among the leading causes of preventable deaths among children under 5 years of age in developing countries. Annual incidence (16%) of diarrheal diseases among children <5 years in Zambia ranks among the top five causes of morbidity and mortality in all ages.^7^

Research shows that antibiotic resistance (ABR) in *Salmonella* is a significant global public health challenge that complicates treatment and contributes to increased morbidity and mortality.^8^  *Salmonella* has shown increasing resistance to commonly used antibiotics such as ampicillin, chloramphenicol and trimethoprim-sulfamethoxazole.^9^ More so, the rise of MDR strains, particularly *Salmonella enterica serovar* Typhi and non-typhoidal *Salmonella*, is associated with severe systemic infections.^8,9^ Misuse and overuse of antibiotics in clinical and veterinary settings are the main factors contributing to the burden of resistant *Salmonella*. The spread of resistant strains through international travel, trade and the food supply chain further amplifies the problem.^10,11^

The growing incidence of *Salmonella* infections in Zambia has been compounded by increasing resistance to commonly used antibiotics such as ampicillin, chloramphenicol and cotrimoxazole.^12^  *Salmonella* MDR strains in both clinical and veterinary settings highlight the role of antibiotic misuse in humans and animals.^9^ This growing resistance threatens the effectiveness of first-line treatments leading to prolonged illness, increased healthcare costs and mortality.^12–14^ Antibiotics constitute the primary treatment for infection caused by *Salmonella* spp. However, because of the widespread use, there has been a rise in AMR to *Salmonella* in recent years.^15^ Essentially, the rise of MDR *Salmonella* strains has highlighted how critical AMR is in public health.

Generally, *Salmonella* infection places a considerable economic strain on healthcare systems.^16^ The resistance to antibiotics makes standard treatments less effective, potentially leading to prolonged illness and undesirable health outcomes, especially in children with weakened immune systems or underlying health issues.^17^ Therefore, this study aimed to analyse patterns of antimicrobial-resistant *Salmonella* spp. in children with AGE at Levy Mwanawasa University Teaching Hospital (LMUTH). In addition, the study aimed analyse the levels of knowledge and practices of the guardians.

## Methods

### Study area

This was a cross-sectional study conducted at LMUTH in children between 0 and 59 months who presented with symptoms of AGE at the outpatient from September 2020 to February 2021. The LMUTH hospital offers specialized services to >1 million people with a bed capacity of 900 and is situated in Lusaka district. The hospital is part of the newly established Levy Mwanawasa Medical University founded as a Public University in 2018.^18^

### Sample size

The study population included children with an episode of diarrhoea as Population One (diarrhoeic) and from the same age category, healthy children attending the <5 years clinic as Population Two (non-diarrhoeic). Children already on antibiotics were not included in the study.

The sample size was calculated using AusvetEpi Tools software,^19^ required to detect a statistically significant difference between two proportions with specified levels of confidence and power. Assuming a 95% confidence level, a statistical power of 80% and a 5% absolute error. In addition, a prevalence of 25.5% of *Salmonella* spp. reported from an earlier study conducted at the University Teaching Hospital,^20^ among children under 5 with diarrheal diseases was assumed. This prevalence was used to calculate the sample size of Population One (diarrhoeic children). It was further assumed that *Salmonella* spp. would be isolated in Population Two (non-diarrhoeic children) at a prevalence of 1%. On the basis of these assumptions, the estimated sample size was 300.

### Sampling

Stool samples of the infants were collected from nappies (fresh stool) using a scoop and placed in stool containers. While from the older children, samples were collected in the sample bottles that were provided. These specimens were then transported in cooler boxes on ice at a temperature of <4°C to the Public Health Laboratory at, University of Zambia for analysis. Unprocessed sample were stored in the fridge at −20°C for 24 hours.

### Epidemiological data

In addition, a questionnaire was administered to parents or guardians to collect epidemiological data and determine the level of knowledge and practices towards *Salmonella* infections.

### Isolations and identification of *Salmonella* species

Using a sterile swab, the stool sample was initially enriched in selenite cystine broth (Oxoid, UK) and aerobically incubated for 24 ± 2 h at 35–37°C. Thereafter, it was subcultured on xylose lysine deoxycholate (XLD) agar (Oxoid, UK) then incubated for 24 ± 2 h at 35–37°C in an aerobic incubator. The XLD plates were examined for presence of colonies suggestive of *Salmonella* i.e. pink colonies with or without black centres. Subsequently, they were purified on nutrient agar/Muller Hinton agar (Oxoid) before subjection to phenotypic characterization. Presumptive *Salmonella* isolates were identified using standard biochemical tests. Triple Sugar Iron agar and Lysine Iron Agar were used for biochemical characterization.

### Serological typing of *Salmonella* isolates

Isolates were categorized into serogroups by using group specific antisera (BD Difco) by assessing the presence of distinct ‘O’ and ‘H’ antigens. Thereafter, antibiotic susceptibility testing was performed.

### Determination of antimicrobial susceptibility patterns

Antimicrobial susceptibility was determined using the Kirby–Bauer disc diffusion method and interpreted according to the CLSI guidelines of 2016.^21^ The antibiotic discs (Oxoid, UK) included sulfamethoxazole/trimethoprim (1.25/23.75 µg), ciprofloxacin (5 µg), cefotaxime (30 µg), tetracycline (30 µg), nalidixic acid (30 µg), cefepime (30 µg), azithromycin (15 µg), imipenem (10 µg) and chloramphenicol (30 µg). The zones of inhibition were grouped into intermediate (I), resistant (R) and susceptible (S) was based on the M100 CLSI2016 guidelines for *Salmonella* species. *Escherichia coli* ATCC 259225922 was used as a quality control strain.

### DNA extraction for *Salmonella* confirmation

*Salmonella* was confirmed by performing PCR assays to detect the *invA* gene specific for *Salmonella*. First, DNA was extracted using the boiling method.^22^ The extracted DNA was then amplified using *Salmonella*-*invA* gene specific primers, namely, S139 (5_GTG AAA TTA TCG CCA CGT TCG GGC AA-3_ and S141 (5_ TCA TCG CAC CGT CAAAGG AAC C-3_). Subsequently, the PCR amplification products were electrophoresed in 1.5% agarose gel stained with ethidium bromide for 40 minutes with a constant voltage of 100 V. Finally, the bands were visualized under ultra-violet light alongside a 100 bp DNA ladder.

### Data management and analysis

All laboratory data were managed in an Excel spreadsheet. Descriptive statistics on socio-demographic characteristics were performed using GraphPad Prism (version 9.0). Antimicrobial susceptibility (AST) data for isolates and resistance gene detection were analysed, and presented as resistance patterns and profiles. AST data were analysed using WHONET 2021. Results for susceptible, intermediate and resistant strains were presented as proportions and graphs.

Further data from the questionnaire interviews were entered in Excel spreadsheets. Mean scores and percentages were used to determine the level of knowledge and practices. Correct answers were scored as one (1) whereas incorrect answers scored zero (0). The knowledge and practices levels were categorized as high, moderate or low on the basis of predetermined thresholds using Bloom’s cut-off criteria.^23^ According to these criteria, scores >60 indicate a high level of knowledge, attitudes and practices, scores between 25 and 59 represent moderate levels and scores <25 suggest a low level.

### Ethical approval

Ethical clearance was sought from Excellence in Research Ethics and Science Converge Institutional Review Board (IRB) committee (IRB Number 00005948), on 31 August 2020. The approval from the National Health Research Authority was obtained (Ref. No. NHRA00010/3/09/2020) and the Provincial and District Health Offices were informed about the study. Consent from guardians was sought in the form of an oral consent script before any sampling was conducted.

## Results

### Socio-demographic characteristics of study participants

Two hundred and five stool samples were collected from children and evaluated for presence of *Salmonella* spp. Out of the 205 samples, 187 (91.2%) were diarrhoeic, of which 17 tested positive for *Salmonella*, while 18 samples (8.8%) were non-diarrhoeic, of which three tested positive for *Salmonella*. Furthermore, of the enrolled 205 participants, 120 (58.5%) were male and 85 (41.5%), were female. Almost all the participants (203 representing 99%) came from urban areas except two. Furthermore, 39 (19.0%) originated from high density areas, 144 (70.2%) from medium density areas and 22 (10.7%) from low density areas (Table 1). The educational levels of the guardians to the participants were largely at secondary school level.

**Table 1.** Socio-demographic characteristics of study participants

Parameter	Level	Frequency	Proportion (%)
Health status	Diarrhoeic	187	91.2
	Non-diarrhoeic	18	8.8
Gender	Male	120	58.5
	Female	85	41.5
Age (months)	0–29	177	86.3
	30–59	28	13.7
Education of guardian	Primary	53	25.9
	Secondary	115	56.1
	Tertiary	37	18.0
Location of household	Urban	203	99
	Rural	2	1
Urban density	High	39	19.0
	Medium	144	70.2
	Low	22	10.7
People per household	0–10	199	97.1
	11–20	6	2.9

### Analysis of the association between *Salmonella* infection of the participants and the potential predictors

There was no association between various predictors and knowledge or practices related to *Salmonella* spp. infection. Specifically, factors such as the participants’ sex (*χ*^2^ = 0.3815, *P* = 0.537), age and likelihood of acquiring infection (*χ*^2^ = 0.03382, *P* = 0.854), guardians’ level of education (*χ*^2^ = 1.003, *P* = 0.606), residence (*χ*^2^ = 3.715, *P* = 0.054), residential density (*χ*^2^ = 3.717, *P* = 0.156) and the number of people per household (*χ*^2^ = 0.3353, *P* = 0.563) showed no significant association (Table 2).

**Table 2.** Summary of univariate analysis between potential risk factors of knowledge and practices towards *Salmonella* infection

Potential risk factors for demography	Frequency	Positive (%)	*P* value
Gender (*n* = 205)			
Male	120	13 (10.83)	0.537
Female	85	7 (8.24)	
Age (months) (*n* = 205)			
0 to 29	177	17 (9.60)	0.854
30 to 59	28	3 (10.71)	
Education of guardian (*n* = 205)			
Primary	53	6	0.606
Secondary	115	12	
Tertiary	37	2	
Location of household (*n* = 205)			
Urban	203	19 (9.36)	0.054
Rural	2	1 (50.00)	
Urban density (*n* = 205)			
High	39	7 (17.95)	0.156
Medium	144	11 (7.64)	
Low	22	2 (9.09)	
People per household (*n* = 205)			
0–10	199	19 (9.55)	0.563
11–20	6	1 (16.67)	

Most guardians did not exhibit knowledge on antibiotics and the meaning of AMR (Table 3). However, a large percentage (72%) of the 205 possessed knowledge on the cause of diarrhoea in children. About 94% of the guardians acknowledged that it was not acceptable to use antibiotics given by friends or family members. Overall, the average knowledge score was moderate.

**Table 3.** Shows potential risk factors for knowledge among the participant’s guardians on *Salmonella* infection

Potential risk factors for knowledge	Frequency	Positive (%)	Score	*P* value
Do you know antibiotics? (*n* = 205)				
Yes	82	6 (7.32)	40^a^	0.337
No	123	14 (11.38)		
Do you know what antibiotic resistance is? (*n* = 205)				
Yes	35	1 (2.86)	17.1^a^	0.131
No	170	19 (11.18)		
Is it acceptable to use antibiotics given by friends or family members as long? (*n* = 205)				
Yes	13	2 (15.38)	93.7^a^	0.480
No	192	18 (9.38)		
Do you know the causes of diarrhea? (*n* = 205)				
Yes	136	11	66.3^a^	0.259
No	69	9		
Average score on knowledge on *Salmonella* infection			54.3^b^	

^a^Proportion considered as knowledge score.

^b^Average knowledge score.

Most of the participants came from households with access to clean water for use in cooking and drinking. However, a good number, 137 (67%), did not treat or filter their water before use. Most households exhibited good practices towards food preparation, disposing of the child’s faeces, food storage, hand washing after use of the toilet and solid waste disposal bearing a good average practice score (69.5%) (Table 4).

**Table 4.** Shows potential risk factors for practices among the participant’s guardians towards *Salmonella* infection

Potential risk factors for practices	Frequency	Positive (%)	Score	*P* value
What is the household’s main source of water for drinking and cooking? (*n* = 205)				
Pipe	116	8 (6.70)	94.6^a^	0.247
Borehole	78	10 (12.82)		
Other	11	2 (18.18)		
Do you treat or filter drinking water? (*n* = 205)				
Yes	68	5 (7.35)	33.2^a^	0.271
No	137	17 (12.41)		
Is there livestock at your household? (*n* = 205)				
Yes	70	6 (8.57)	65.6^a^	0.681
No	135	14 (10.37)		
Is the child breast feeding? (*n* = 205)				
Yes	93	11 (11.83)	54.6^a^	0.362
No	112	9 (8.04)		
Do you wash your hands before preparing or giving food to the child? (*n* = 205)				
Yes	174	16 (9.20)	84.9^a^	0.522
No	31	4 (12.90)		
Do you wash hands after disposing off the child's feces? (*n* = 205)				
Yes	178	15 (8.43)	86.8^a^	0.100
No	27	5 (18.52)		
Where do you normally store the prepared food for the children? (*n* = 205)				
Store correctly	148	14 (9.46)	72.2^a^	0.818
Do not store	57	6 (10.53)		
What do you use to feed the child? (*n* = 205)				
Spoon	9	2 (22.22)	4.4^a^	0.197
Hands/fingers/breastfeeding	196	18 (9.18)		
What toilet facility do you have? (*n* = 205)				
Pit latrine/Flushable toilet	202	19 (9.41)	98.5^a^	0.166
No facility	3	1 (33.33)		
Do you wash hands after going to the toilet? (*n* = 205)				
Yes	193	18 (9.33)	94.1^a^	0.406
No	12	2 (16.67)		
How do you dispose of your solid waste? (*n* = 205)				
Bin/Pit	155	15 (9.68)	75.6^a^	0.947
Roadside/Openly	50	5 (10.00)		
Average score on practice towards *Salmonella* infection			69.5^b^	

^a^Proportion considered as practice score.

^b^Average practice score.

### Serotyping

After identification from bacterial culture and biochemical tests, a total of 28 isolates were subjected to *Salmonella* serotyping using anti-O and anti-H antisera. Out of these, 14 isolates (50%) were positive for both anti-O and anti-H antisera, whereas two isolates (7.1%) were negative for both anti-O and anti-H antisera. Finally, six isolates (21.4%) were anti-O positive, anti-H negative while another six isolates (21.4%) were anti-O negative, anti-H positive.

### Antimicrobial susceptibility patterns of *Salmonella* spp.

**Figure 1.**
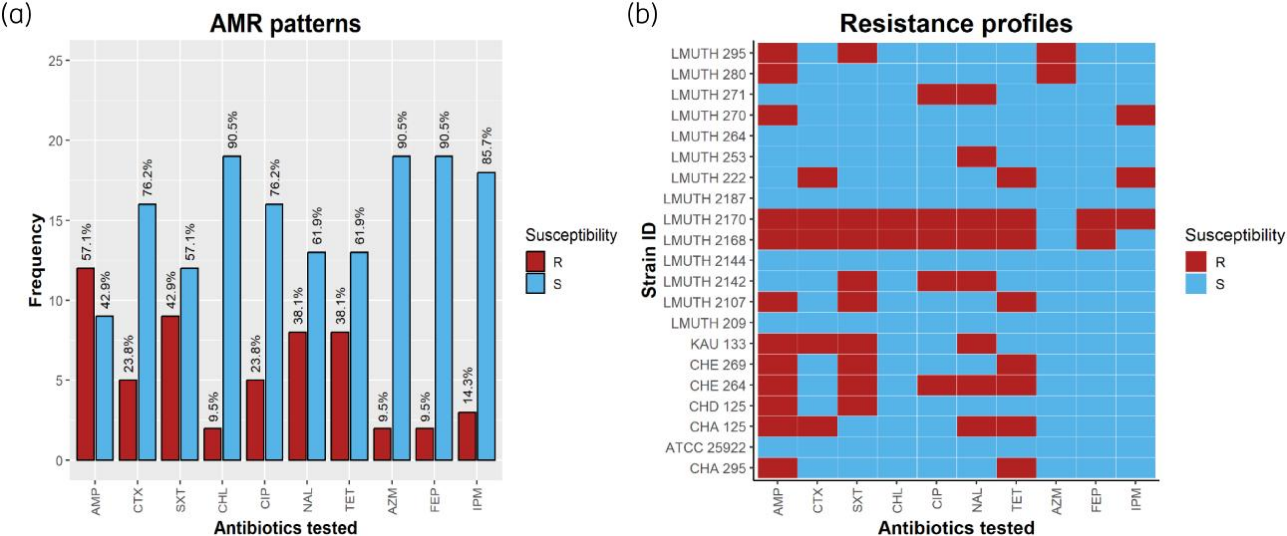
Antimicrobial susceptibility patterns (a) and resistance profiles (b) of *Salmonella* spp. from stool. Abbreviations: AZM, azithromycin; CHL, chloramphenicol; CIP, ciprofloxacin; CTX, cefotaxime; FEP, cefepime; IPM, imipenem; NAL, nalidixic acid; SXT, trimethoprim-sulfamethoxazole; TET, tetracycline (key: R, resistant; S, susceptible). Frequency on the *y*-axis represents the number of isolates, and percentages on the bars represent the proportion of susceptibility.

The AST patterns of *Salmonella* spp. in stool samples are illustrated in Figure 1. Most of the isolates (42.9%) showed resistance to trimethoprim-sulfamethoxazole. However, the isolates were largely susceptible to the remaining eight antibiotics tested. According to the results of this study, *Salmonella* spp. exhibited the highest susceptibility to chloramphenicol, cefepime and azithromycin (90.5%), followed by imipenem (85.7%), ciprofloxacin and cefotaxime (both at 76.2%). Nalidixic acid and tetracycline were at 61.9%. Out of 20 *Salmonella* isolates identified, only four were susceptible to all tested antibiotics.

### *Salmonella* spp. identification

The *Salmonella* spp. was confirmed by PCR using the *invA*gene (Figure S1, available as Supplementary data at *JAC-AMR* Online). Out of the initial 205 samples, 20 (9.8%) were confirmed *Salmonella* positive using PCR.

## Discussion

This study aimed to analyse patterns of the antimicrobial-resistant *Salmonella* spp. in children <5 years as well as knowledge and practices of their guardians. This study target to sample 300 children, however, we only managed to sample 205 because it was difficult to access patients, and hospital avoidance by patients was common during the COVID-19 pandemic^24^ when the study was carried out. The prevalence of *Salmonella* infection was observed to be low compared with what has been observed in other studies, albeit the sample size of the current study was lower.^25,20^ The low prevalence could be possibly due to various factors such as location of the household, as most children came from urban areas where hygienic practices tend to be good. Nonetheless, the diarrhoeic population seemed to have more positive *Salmonella* infections compared with the non-diarrhoeic population. This observation might have been due to the smaller number of non-diarrhoeic children included in the study. It may also be influenced by the role of asymptomatic carriers in *Salmonella* transmission. As healthy individuals are ideally expected to have either no *Salmonella* or a very low prevalence, asymptomatic carriers can still harbour the pathogen and contribute to its spread without showing clinical symptoms.^15^

*Salmonella* infections are prevalent in communities that usually have low hygiene status and households that have meals prone to contamination.^26^ This is because *Salmonella* contamination of food is a major problem in such communities.^27,20^ The inadequate WASH strategies coming from limited water access, poor environmental and hygiene, lack of separate, private and secure toilets and washing facilities in LMICs still highlight the need for more interventions.^28^

About 66.8% of the participants did not treat or filter water before drinking, which posed a risk of acquiring *Salmonella* infection.^29,30^ Furthermore, with about 95.6% of mothers/guardians using hands to feed their children, the likelihood of infection was high since hands normally carry bacteria.^31^ There is a need for safer feeding habits and behavioural change among guardians/caregivers to reduce this risk of *Salmonella* infections.^32^

Most of the respondents (66.3%) were familiar with the cause of *Salmonella* infection. This corroborates with findings from another study with similar observations on the participants’ knowledge on the major ways of transmission.^33^ Of the respondents, 93.7% indicated that it was not acceptable to use antibiotics given by friends or family. Despite sufficient access to reliable information sources, the 7.3% gap indicates that there is still need for awareness on antimicrobial use and gene transfer of AMR genes to humans that comes with the misuse of antibiotics. Initiatives such as antimicrobial stewardships using the One-Health framework that accounts for all transmission points are also very important to curb AMR.^34^ This is withstanding the findings of this study showing a significant understanding about the disease as seen from the moderate knowledge score (54.3%) similar to earlier studies (57.8%).^35^

The observed 69.5% practice score on good infection-prevention practices was higher than only 42.3% observed elsewhere.^36^ The good average score for practices related to *Salmonella* infections indicates good adherence to preventive measures. Besides, studies have reported that good practices, for example, hand washing, show a general common understanding of health risks.^37^

The resistance patterns observed in this study is similar to the findings in observed in others studies, which reported *Salmonella* spp. being mostly resistant to nalidixic acid and tetracyclines.^38^ Previous research has also reported *Salmonella* spp. to be resistant to chloramphenicol, sulfamethoxazole, tetracycline and trimethoprim.^39^ There is a need for enhanced approaches for AMR surveillance on *Salmonella* spp. due to its resistance to multiple antibiotics,^40^ as seen in this study.

If humans become infected with resistant *Salmonella*, it becomes difficult to treat such infections. This becomes a public health burden as it limits drug treatment options and may result in increased disease burdens.

This study was conducted at a single hospital in Zambia. This may limit the extrapolation of the findings to other larger regions or healthcare settings. This prompts additional studies with a larger study area and sample size to better understand the risk factors contributing to *Salmonella* spp. infections and antibiotic resistance in children <5 years. There may also have been some bias related to self-reporting and recall.

### Conclusion

We observed a low prevalence of *Salmonella* infection in children <5 years at LMUTH. Out of the identified 20 isolates of *Salmonella* spp., only four isolates were susceptible to all the drugs used. Finally, the potential risk factors for acquiring *Salmonella* spp. identified in this study include the failure to treat or filter drinking water and guardians’ poor feeding habits with their children.

## Supplementary Material

dlag031_Supplementary_Data

## Supplementary data

Figure S1 is available as Supplementary data at *JAC-AMR* Online.
