# Supplementary material for: Antimicrobial resistance, characterization, and knowledge practices of Salmonella spp. infection in under-five children with acute gastroenteritis at Levy Mwanawasa University Teaching Hospital, Lusaka Zambia
Source: JAC Antimicrob Resist. 2026 Mar 19;8(2):dlag031. doi: 10.1093/jacamr/dlag031 (PMC13001811; doi:10.1093/jacamr/dlag031)
Supplement: dlag031_Supplementary_Data [file dlag031_supplementary_data.docx]

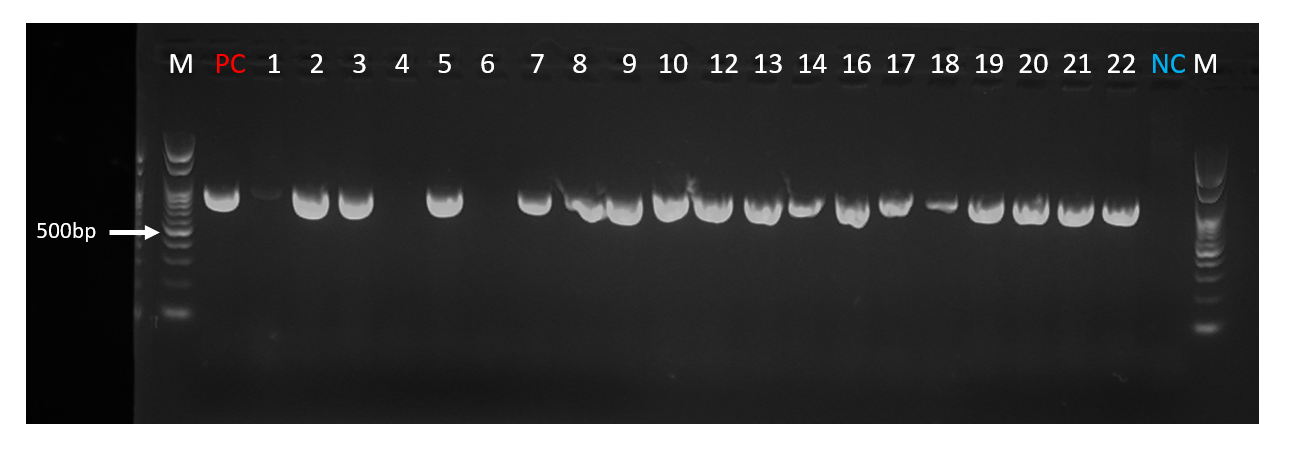


**Figure S1.**Agarose gel electrophoresis analysis of PCR amplification of bacterial genomic DNA extracted from the Salmonella isolates
